# Supplementary material for: Quantum Capacitance of a Topological Insulator-Ferromagnet Interface
Source: Sci Rep. 2017 Mar 24;7:45016. doi: 10.1038/srep45016 (PMC5364463; doi:10.1038/srep45016)
Supplement: Supplementary Information [file srep45016-s1.pdf]

Quantum Capacitance of a Topological Insulator-Ferromagnet Interface

Zhuo Bin Siu [1], Debashree Chowdhury* [2], Mansoor B.A. Jalil [1], and Banasri Basu [3]
[1] Computational Nanoelectronics and Nanodevices Laboratory, National University of Singapore, Singapore
[2] Department of Physics, Harish-Chandra Research institute, Chhatnag Road, Jhusi, Allahabad, U. P. 211019,India
[3] Physics and Applied Mathematics Unit, Indian Statistical Institute, Kolkata 700108, India
[*] Corresponding author : debashreephys@gmail.com

Calculation of Density of states

The DOS can be expressed in terms of self energy as

$$D(E) = \mathrm{Im}[\frac{\Sigma^-(E)}{\pi^2 l^2}\Gamma_0^2],$$

where $\Sigma^-(E) = \Gamma_0^2 \sum_n \sum_{\alpha,s} \frac{1}{E-E^T_{n\alpha s}-\Sigma^-(E)} = \sum_{\alpha,s}\Sigma^-_{\alpha,s}(E)$, is the self energy of the system with Γ_0 as the impurity induced LL broadening. The modified energy of the system is $E^T_{n\alpha s} = E_{n\alpha s} + E^{(2)}_{n\alpha s}$, where $E^{(2)}_{n\alpha s} \equiv E^{2(p)}_{n\alpha s} + E^{2(m)}_{n\alpha s}$. As $E^{(2)}_n << E_n$ (we omit the indices α and s when no confusion might arise), we have, for each value of α and s,

$$\Sigma^-_{\alpha,s}(E) = \Gamma_0^2 \sum_n \frac{1}{E - E_{n,\alpha,s} - \Sigma^-(E)} + \Gamma_0^2 \sum_n \frac{E^{(2)}_{n\alpha s}}{[E - E_{n,\alpha,s} - \Sigma^-(E)]^2},$$

on Taylor expansion. The first term is easy to calculate and we finally can obtain

$$\Gamma_0^2 \sum_n \frac{1}{E - E_{n\alpha s} - \Sigma^-(E)} = \frac{\pi\Gamma_0^2 E}{w_c^2}\cot(\pi n_{0,s})$$

where $n_{0,s} = \frac{1}{(2\hbar\omega_c^2)}[\{E - (\Delta + s\Delta_t)^2]$ is the pole neglecting the contribution of the self-energy. Note that $n_{0,s}$ has the same value regardless of whether $\alpha = 0$ or 1 so we have omitted the α index in n_{0s}.

The second term in the self energy expression contains a repeated pole and using standard summation formula we have, temporarily dropping the α and s indices for notational brevity,

$$\Sigma^-(E)_{\alpha,s\text{2nd term}} = -\frac{\pi\Gamma_0^2}{\omega_c^4}\left(\omega_c^2 \cot(\pi n_0)E^{(2)}_{n_0} - \pi\csc(\pi n_0)^2 (E_{n_0})^2 E^{(2)}_{n_0} + \cot(\pi n_0)(E_{n_0})^2(\partial_z E^{(2)}_{n_0})\right) \quad (1)$$

Noting that $E \ll 1$, neglecting higher order terms in E_{n_0} , lands us at

$$\Sigma^-_{\alpha,s}(E) = \frac{\pi\Gamma_0^2 E}{\omega_c^2}\left(1 - \frac{E^{(2)}_{n_{0,s}\alpha s}}{\omega_c^2}\right)\cot \pi n_{0,s}. \quad (2)$$

Within the $E^2_{n_0}$, by summing over each s and α branch we can, following the method of Refs. [?] and [?] extract the total DOS as

$$D(E) = \sum_{s=\pm1,\alpha} \frac{D_0(E)}{2}\left[1 + 2\sum_{u=1}^{\infty} \exp\{-u(\frac{\pi\Gamma_0 E}{\omega_c^2})^2\}\cos\left(u\frac{\pi}{\omega_c^2}(E^2 - (\Delta + s\Delta_t)^2)\right)\right]\left(1 - \frac{E^{(2)}_{n_{0,s}\alpha s}}{\omega_c^2}\right) \quad (3)$$

where $D_0(E) = \frac{2E}{\pi\omega_c^2}$, $n_{0,s} = \frac{1}{\omega_c^2}\left[E^2 - (\Delta + s\Delta_t)^2\right]$. We've recovered the asymptotic form $(n > 1)$ of the DOS with a minute additional term which manifests due to warping and in-plane magnetization.

One can further rewrite the DOS by expressing the summation on the right hand side as $\mathrm{Re}\sum_{u=1,\infty}(\exp(u(-\gamma^2 + i\beta))$ where $\gamma = (\frac{\pi\Gamma_0 E}{\omega_c^2})$ and $\beta = \frac{\pi}{\omega_c^2}(E^2 - (\Delta + s\Delta_t)^2)$. Evaluating the sum gives

$$\mathrm{Re}\sum_{u=1}^{\infty}(\exp(u(-\gamma^2 + i\beta)) = \frac{\exp(-\gamma^2)(\cos\beta - \exp(-\gamma^2))}{1 - 2\cos\beta\exp(-\gamma^2) + \exp(-2\gamma^2)}.$$

The DOS can then be written as

$$D(E) = \sum_{s,\alpha} D(E,s,\alpha) \tag{4}$$

where

$$D(E,s,\alpha) = \frac{D_0(E)}{2} \frac{\exp(2(\frac{\pi\Gamma_0 E}{\omega_c^2})^2)\cos(\pi\frac{[E^2-(\Delta+s\Delta_t)^2]}{\omega_c^2}) - 1}{1 - 2\exp\{(\frac{\pi\Gamma_0 E}{\omega_c^2})^2\}\cos(\pi\frac{[E^2-(\Delta+s\Delta_t)^2]}{\omega_c^2}) + \exp\{2(\frac{\pi\Gamma_0 E}{\omega_c^2})^2\}} \left(1 - \frac{E^{(2)}_{n_{0,s}\alpha s}}{\omega_c^2}\right). \tag{5}$$
